# Global Re-Analysis Confirms Absence of the *DNAJB1*::*PRKACA* Fusion in Hepatoblastoma. Comment on Fleifil et al. DNAJB1-PKAc Kinase Is Expressed in Young Patients with Pediatric Liver Cancers and Enhances Carcinogenic Pathways. *Cancers* 2025, *17*, 83

**DOI:** 10.3390/cancers18050877

**Published:** 2026-03-09

**Authors:** Waqar Arif, Sanford M. Simon, Allison F. O’Neill, Juan Putra, Dolores H. López-Terrada, Mark Yarchoan, Jessica Zucman-Rossi, Theo Z. Hirsch

**Affiliations:** 1 Department of Oncology, Sidney Kimmel Comprehensive Cancer Center, Johns Hopkins University School of Medicine, Baltimore, MD 21231, USA; warif1@jhu.edu (W.A.); mark.yarchoan@jhmi.edu (M.Y.); 2 Laboratory of Cellular Biophysics, The Rockefeller University, New York, NY 10065, USA; simon@rockefeller.edu; 3 Department of Pediatric Oncology, Dana-Farber Cancer Institute, Harvard Medical School, Boston, MA 02115, USA; allison_oneill@dfci.harvard.edu; 4 Department of Pathology, Boston Children’s Hospital, Boston, MA 02115, USA; juan.putra@childrens.harvard.edu; 5 Department of Pathology, Texas Children’s Hospital, Houston, TX 77030, USA; dhterrad@texaschildrens.org; 6 Centre de Recherche des Cordeliers, Université Paris Cité, Sorbonne Université, INSERM, 75006 Paris, France; jessica.zucman-rossi@inserm.fr; 7 Hôpital Européen Georges Pompidou, Assistance Publique-Hôpitaux de Paris, APHP, 75015 Paris, France

## Abstract

Fleifil et al. recently reported the *DNAJB1*::*PRKACA* fusion in 70% of hepatoblastomas (HB), suggesting its diagnostic relevance beyond fibrolamellar carcinoma (FLC). Here, an international consortium re-examined this claim using 225 independent assays across 148 HB samples from five institutions. We found no evidence of the *DNAJB1*::*PRKACA* fusion in any HB case, in contrast to 286/290 FLC samples. These findings demonstrate that the fusion remains specific to FLC and underscore the need for rigorous validation before clinical interpretation of molecular findings in pediatric liver tumors.

## Simple Summary

The *DNAJB1*::*PRKACA* gene fusion is a well-established marker of fibrolamellar carcinoma, a rare liver cancer. A recent study reported that this fusion is also present in most hepatoblastomas, the most common liver cancer in children, which would have important diagnostic implications. To reassess this claim, we analyzed 148 hepatoblastoma samples using multiple independent and rigorous methods. We found no evidence of the fusion in any case, while it remained consistently detectable in fibrolamellar carcinoma. These results confirm that *DNAJB1*::*PRKACA* is specific to fibrolamellar carcinoma.

Fibrolamellar carcinoma (FLC) is a rare liver cancer affecting adolescents and young adults. Diagnosis initially relied upon specific histological features, including the presence of oncocytic tumor cells and intratumoral fibrosis arranged in a lamellar fashion [1]. Our understanding of FLC biology was accelerated by the identification of a recurrent transcript fusion, *DNAJB1*::*PRKACA*, resulting from a ~400 kb deletion on chromosome 19. The hallmark study identifying this fusion demonstrated its presence in 100% of tumors in a cohort of 14 FLC patients [2]. Follow-up analyses have indicated that this fusion was pathognomonic of FLC among all other liver tumors, including a large number of hepatocellular carcinoma (HCC), cholangiocarcinoma, hepatocellular adenoma, and hepatoblastoma [3,4]. In other organs, the *DNAJB1*::*PRKACA* fusion was only identified in 26% of intraductal oncocytic papillary neoplasms of the pancreas and bile duct, a rare entity [5]. Conversely, tumors diagnosed as FLC in patients older than 40 years old, which often show mixed HCC/FLC histological features, were found to lack the fusion [6]. It was later shown that these tumors are instead more consistent with BAP1-HCC, a group of tumors driven by biallelic loss of the *BAP1* tumor suppressor, representing ~5% of adult HCC cases [7]. BAP1-HCC activates protein kinase A through the combined loss of its regulatory subunit *PRKAR2A* and gains/amplifications of its catalytic subunit *PRKACA*, as opposed to deletion leading to the *DNAJB1*::*PRKACA* fusion in younger onset FLC [7]. Rare reports of FLC in patients with Carney complex, in the absence of the *DNAJB1*::*PRKACA* fusion but with the loss of another regulatory subunit, *PRKAR1A*, further highlight the driving role of PKA activation in at least some tumors with fibrolamellar features [8].

We read with great interest the article by Fleifil et al. (*Cancers* 2024, *17*, 83) [9], which reports the presence of the *DNAJB1*::*PRKACA* fusion in 70% of hepatoblastomas (HB) and even in adjacent non-tumor tissue. This claim is based on three methods: (i) the presence of an extra band on Western blot with an antibody targeting native PKA, compatible with the DNAJB1-PKAc fusion protein; (ii) qRT-PCR to detect the fusion transcript; and (iii) a positive signal with an in-house fusion-specific antibody. While this result is intriguing, it contradicts all previous reports. Furthermore, despite having RNA sequencing (RNAseq) data from some fusion-positive HB samples, the authors did not use RNAseq-based fusion detection tools to validate the presence of the fusion in HB patients, even though this is the current gold standard for transcript fusion detection.

The lack of validation in an external cohort in Feifil et al. prompted us to assemble an international working group of five institutions in the USA and France, all experts in genomics of pediatric liver tumors. We routinely analyze FLC and HB samples for research and/or clinical purposes. We independently reviewed data generated in our different institutions, utilizing multiple technical and analytical experiments for fusion detection at the DNA and RNA levels (whole-genome sequencing, bulk RNAseq, RT-PCR with specific probes, Sanger sequencing), most of which have been previously published [2,7,8,10,11,12,13,14,15,16]. Institutional and ethical review board approvals were obtained at each institution for this work. In a de-identified fashion, we collaboratively shared our results within this global team of investigators.

In a total of 148 different HB samples analyzed in 225 independent experiments, we confirm the complete absence of the *DNAJB1*::*PRKACA* (0/148), giving an upper limit of 2.5% for the prevalence of the fusion in HB. We can thus reject the claim by Fleifil et al. of a 70% prevalence of the fusion in HB (binomial test, *p* < 2.2 × 10^−16^). Table 1 shows the breakdown of our analyses by group and by method, highlighting the concordance of our results. For seven HB cases, the absence of the *DNAJB1*::*PRKACA* fusion was confirmed across three spatially distinct tumor regions. We further emphasize the use of the same analytical pipeline to detect the *DNAJB1*::*PRKACA* fusion in 286/290 FLC samples and to confirm its absence in HB samples, highlighting the sensitivity of these methods. We may add that FLC samples have typically lower tumor purity than HB samples due to the abundant stromal cells composing the fibrolamellar features [15], as well as normal and immune cells, excluding a possible lack of sensitivity in HB.

In summary, our comprehensive analyses across five expert centers conclusively reject the hypothesis of a prevalent *DNAJB1*::*PRKACA* fusion in HB. This conclusion is supported by 225 independent assays showing a complete absence of the fusion in 148 HB samples, despite consistent detection in 199/203 FLC cases. We reaffirm that *DNAJB1*::*PRKACA* remains specific to fibrolamellar carcinoma among liver tumors and caution against its use as a diagnostic marker for hepatoblastoma without rigorous validation in external cohorts. We chose to comment on this issue to avoid confusion about the diagnostic significance of the *DNAJB1*::*PRKACA* fusion in pediatric liver tumors.

## Figures and Tables

**Table 1:** Multi-institutional, multi-method detection of the *DNAJB1*::*PRKACA* fusion in HB and FLC samples. Each row corresponds to one detection method applied by a single research group. The same method was used to assess both HB and FLC samples. For the overall analysis, we summed non-overlapping HB and FLC samples.

Group	Experimental Method	*DNAJB1*::*PRKACA* Fusion in HB	*DNAJB1*::*PRKACA* Fusion in FLC	Associated Publications
Zucman-Rossi, Cordeliers Research Center, France	Bulk RNAseq, TopHat Fusion	0/100	20/20	[7,13]
Zucman-Rossi, Cordeliers Research Center, France	Whole-genome sequencing, structural variant detection using Manta	0/48	4/4	[13]
López-Terrada, Baylor College of Medicine, USA	RT-PCR + Sanger sequencing	0/18	22/22	[12]
López-Terrada, Baylor College of Medicine, USA	Bulk RNAseq, deFuse (v.0.6.1.), followed by manual review and confirmation	0/20	8/8	[11]
O’Neill, Dana Farber, USA	Archer FusionPlex Assay	0/10	8/8	
Yarchoan, Johns Hopkins, USA	RT-PCR, including three spatially distinct tumor regions for n = 7 HB cases	0/7	3/3	
Simon, Rockefeller University, USA	Dual RT-PCR from the first exon of *DNAJB1* to the second exon of *PRKACA*	0/11	75/75	[2,10,14,15,16]
Simon, Rockefeller University, USA	Bulk RNAseq, reads mapping to both the first exon of *DNAJB1* and the second exon of *PRKACA*	0/11	148/152 *	[2,8,10,14,15,16]
**Overall, by experiment**	**Count of all independent experiments**	**0/225**	286/290 *	
**Overall, by independent sample**	**Count of non-overlapping samples**	**0/148**	199/203 *	

* Four samples with a histological diagnosis of FLC did not harbor the *DNAJB1*::*PRKACA* fusion but instead presented a biallelic loss of *PRKAR1A* [8].

## Data Availability

The data presented in this Comment originate from previous publications as detailed in Table 1.

## Abbreviations

The following abbreviations are used in this manuscript:

FLC	Fibrolamellar carcinoma
HB	Hepatoblastoma
HCC	Hepatocellular carcinoma
PKA	Protein kinase A
RT-PCR	Reverse transcription polymerase chain reaction
